# Expression of *WNT* Family Genes in Mesenchymal Stromal Cells of the Hematopoietic Niche in Patients with Different Responses to Multiple Myeloma Treatment

**DOI:** 10.3390/ijms26136236

**Published:** 2025-06-27

**Authors:** Liubov A. Belik, Natella I. Enukashvily, Natalia Y. Semenova, Dmitrii I. Ostromyshenskii, Ekaterina V. Motyko, Anna N. Kirienko, Daria V. Kustova, Stanislav S. Bessmeltsev, Sergey V. Sidorkevich, Irina S. Martynkevich

**Affiliations:** 1 Laboratory of the Non-Coding DNA Study, Institute of Cytology, Russian Academy of Sciences, 194064 St. Petersburg, Russia; 2 Russian Research Institute of Hematology and Transfusiology, Federal Medical-Biological Agency of Russia of Russia, 1910243 St. Petersburg, Russiamis2907@mail.ru (I.S.M.); 3 Cell Technologies Center Pokrovsky, 199106 St. Petersburg, Russia; 4 Cell Technologies Laboratory, North-Western State Medical University Named After I. I. Mechnikov, 191015 St. Petersburg, Russia

**Keywords:** WNT signaling pathway, multiple myeloma, mesenchymal stromal cells, tumor microenvironment, WNT3A, WNT5A, WNT10B, β-catenin

## Abstract

Mesenchymal stromal cells of the tumor microenvironment (TME) play a significant role in the progression of multiple myeloma (MM). The cells of the TME demonstrate resistance to treatment, thereby creating a favorable environment for disease relapse. The status of the TME during remission is poorly understood. An association between treatment response and TME status (including signaling pathways) has been suggested. One of the key players in the establishment of the MM TME is WNT signaling. In this study, we evaluated the expression of WNT family proteins in the TME and MM cells to assess their potential as TME markers and predictors of treatment response. A bioinformatic analysis of normal and malignant plasma cells, combined with an analysis of published data, revealed the following differentially expressed WNT genes: *WNT5A*, *WNT10B*, *CTNNB1*, and *WNT3A*. Immunohistochemical staining with the antibodies against the proteins encoded by the genes was conducted on trephine biopsy samples of bone marrow from healthy donors and patients with different responses to therapy. A quantitative analysis of the immunohistochemical data revealed differences in the amounts of WNT3A, WNT5A, WNT10B, and β-catenin proteins in the bone marrow before treatment depending on the subsequent responses of the patients to therapy. Multiplex fluorescent immunohistochemical staining with tyramide signal amplification revealed that WNT3A was predominantly present in mesenchymal stromal cells, whereas WNT5A and WNT10B were primarily observed in plasma cells. β-catenin was detected in both cell types. We analyzed the mRNA levels of the *WNT* gene family and *CTNNB1* in MSC cultures from healthy donors and patients using qPCR. These genes were differentially expressed in MSC cultures derived from patients and healthy donors, as well as between patients grouped according to their response to therapy. Therefore, WNT proteins and β-catenin can be considered potential markers to assess the state of the tumor niche.

## 1. Introduction

Multiple myeloma (MM) is a plasma cell (PC) malignancy. With an incidence of 4.5–6 per 100,000 per year in high-income countries, it accounts for 10–13% (2nd) of all hematological cancers [1,2]. Patients with MM usually suffer hypercalcemia, renal failure, anemia, and osteolytic bone lesions. While there are therapeutic options to induce a temporary remission, MM remains an incurable disease [3].

Most of MM patients experience several relapses of the disease. One of the most critical factors in the survival of MM cells is their ability to resist the effects of drugs used to treat the disease [4]. A major contributor to resistance is the tumor microenvironment (TME), which consists of a variety of cell types, including osteoblasts, osteoclasts, adipocytes, endothelium, immune cells, and mesenchymal stromal cells [5,6]. Mesenchymal stromal cells (MSCs) are an important cellular component of the niche [5,7]. MSCs regulate the activity of hematopoietic stem cells (HSCs) and participate in bone tissue remodeling by differentiating into osteoblasts [8,9,10]. In MM bone marrow (BM), MSCs acquire a tumor-associated phenotype that promotes tumor growth, drug resistance [11,12,13,14], and bone resorption [15].

The complex interaction between tumor cells and the TME plays an important role in MM development [16]. These interactions are mediated by numerous signaling cascades that are dysregulated during the development of MM (e.g., RAS/MAPK, PI3K/AKT/mTOR, NF-κB, WNT) [17,18,19]. The WNT pathway is involved in the maintenance of normal hematopoietic niche. It regulates the balance between osteoblasts and osteoclasts, supporting bone tissue renewal, and it is also responsible for cell proliferation, differentiation, and other processes [20,21,22,23,24]. In MM, activation of the WNT pathway in tumor cells promotes their proliferation [25], while its suppression in the TME by inhibitors secreted by tumor cells leads to impaired osteogenesis [26,27]. Osteolysis in MM is due to the suppression of canonical WNT signaling caused by DKK1, a soluble inhibitor of this pathway secreted by MM cells [26]. Anti-MM drugs have different effects on osteogenesis. Bortezomib increases osteogenesis via the WNT-independent activation of β-catenin/TCF signaling; while osteonecrosis is a recognized toxicity associated with bisphosphonate use, denosumab, a RANKL-neutralizing antibody, is given to patients with renal failure (a complication of MM) but increases the risk of osteonecrosis [28].

The WNT cascades are activated by the binding of protein WNT ligands with receptors on the cell surface. In humans, the WNT ligands are now known to be a group of 19 WNT glycoproteins [29]. MSCs respond to WNT signaling and, in their turn, secrete ligands [30,31]. In the canonical WNT pathway, the intracellular mediator is the β-catenin protein (encoded by the *CTNNB1* gene). WNT ligands induce the dissociation of the β-catenin “destruction complex,” which leads to the translocation of free cytoplasmic β-catenin into the nucleus. There, it binds specifically to TCF/LEF (T-cell factor/lymphoid enhancer factor) transcription factors [32]. Approximately 85% of β-catenin targets are mediated by its interaction with these transcription factors. However, there are also TCF/LEF-independent β-catenin effector pathways, e.g., FOXO4 [33]. Non-canonical WNT cascades involve other intracellular proteins [21]. Alterations in the function of the WNT/β-catenin pathway have been observed in many cancers. These changes are often the result of mutations in genes encoding components of the pathway [34]. However, during carcinogenesis, the WNT pathway can be dysregulated not only by genetic mutations. The role of epigenetic modifications has been demonstrated; in some hematological neoplasia, including MM, the concurrent methylation of multiple WNT inhibitors has been observed [21,35]. According to the data published in [36], almost half of the enrolled patients had hypermethylated WIF1, Dkk3, APC and sFRP1, sFRP2, sFRP4, and sFRP5 promoter regions. Thus, aberrant epigenetics play an important role in the aberrant activation of the WNT pathways and, hence, MM progression. In MM, these changes in the WNT pathway may have other causes, including changes in the levels of WNT proteins in the TME [21]. Moreover, MM cells disrupt WNT signaling in the BM by secreting WNT antagonists. On the other hand, the WNT pathway is over-activated within the tumor cells themselves, leading to an increase in their proliferation and migration and presumably enhancing drug resistance [21]. Stimulation occurs in a paracrine way, as a result of the TME-derived WNT proteins, or in an autocrine way by the canonical ligands that are expressed by MM cells themselves or combinations thereof [21]. Thus, the source of WNT ligands for MM cells may be the cells themselves (autocrine stimulation), but the cells of the TME may also be a source of secreted ligands (paracrine regulation) [21]. As MSCs and MM cells interact closely [37], changes in WNT levels in tumor-associated MSCs can be expected. The interaction of MM cells with the TME is an important factor in tumor progression and the development of drug resistance [37]. The recovery of WNT pathway characteristics after the treatment of hematological malignancies is currently under study. There is evidence that alterations associated with MM development in the BM niche persist after treatment [13,38]. In routine clinical practice, MM is diagnosed by a limited number of methods, including the histological examination of BM trephine biopsies [39]. This method assesses both BM lesions and infiltration; however, there are currently no established clinical guidelines to evaluate the MM TME, whether it is normal or cancer-associated. It is, therefore, essential to identify biomarkers that can indicate the state of the TME.

The objective of this study was to examine the expression of WNT proteins in MM BM in order to identify potential biomarkers that can be utilized to detect the stromal component of the TME and to predict the response to therapy.

## 2. Results

### 2.1. Differential Expression of WNT Family Genes and CTNNB1 Gene in the PCs of MM Patients

**Figure 1:**
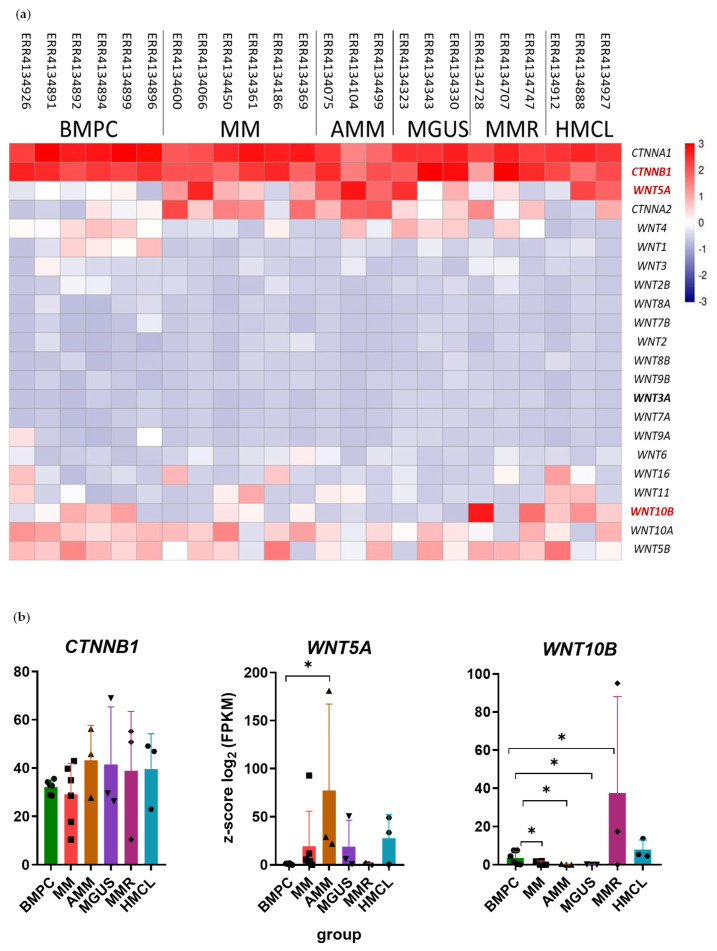
(**a**) Differential expression of *WNT* family genes and *CTNNA1*, *CTNNA2*, and *CTNNB1* genes. Run accession numbers in the SRA database are shown above. The color scale shows z-score values calculated for each gene (shown on the right); (**b**) differential expression of *WNT5A*, *WNT10B*, and *CTNNB1* genes plotted as bars with individual values (shown as dots). Data represented as mean ± SD. * *p* < 0.05. The Y-axis shows z-score values. The X-axis—groups of samples (BMPC—healthy BM PCs; MM—symptomatic MM; AMM—asymptomatic MM; MGUS—monoclonal gammopathy of undetermined significance; MMR—relapsed MM; HMCL—human myeloma cell lines).

To select potential biomarkers of the TME, the expression of *WNT* family genes and catenin genes (*CTNNA1*, *CTNNA2*, *CTNNB*) was first evaluated in silico. Differential gene expression (DGE) analysis was performed using reads obtained from sequencing cDNA from BM samples of pre-treated patients with various forms of MM and monoclonal gammopathy of undetermined significance (MGUS) (Figure 1).

Expression of the *CTNNB1* gene encoding β-catenin, a key intracellular mediator in the canonical WNT signaling pathway, was different in a fraction of MM and MGUS patients compared to PCs from healthy BM (Figure 1a,b). Among the *WNT* family genes, *WNT5A* and *WNT10B* genes were differentially expressed. *WNT5A* expression was significantly upregulated in patients with asymptomatic MM (AMM). A trend of increase in WNT5A expression was also observed in other forms of MM, MGUS, and MM cell lines (HMCL). *WNT10B* expression was upregulated in most samples of relapsed MM and in MM cell lines, while it decreased in MM, AMM, and MGUS samples (Figure 1a,b). Changes in the *WNT1*, *WNT10A* (downregulated), and *WNT5B* (upregulated/downregulated) expression were also observed (Figure 1a).

### 2.2. Quantification of WNT and β-Catenin Protein Levels in the BM of MM Patients

After the analysis of expression changes in PC transcriptomes, the next step was to examine the distribution of proteins within the BM niche in patient trephine biopsies and to compare it with their distribution in HD trephines. WNT3A, WNT5A, WNT10B, and β-catenin proteins were selected based on bioinformatics analysis (Figure 1), and WNT3A was added according to the published data [25,40,41].

**Figure 2:**
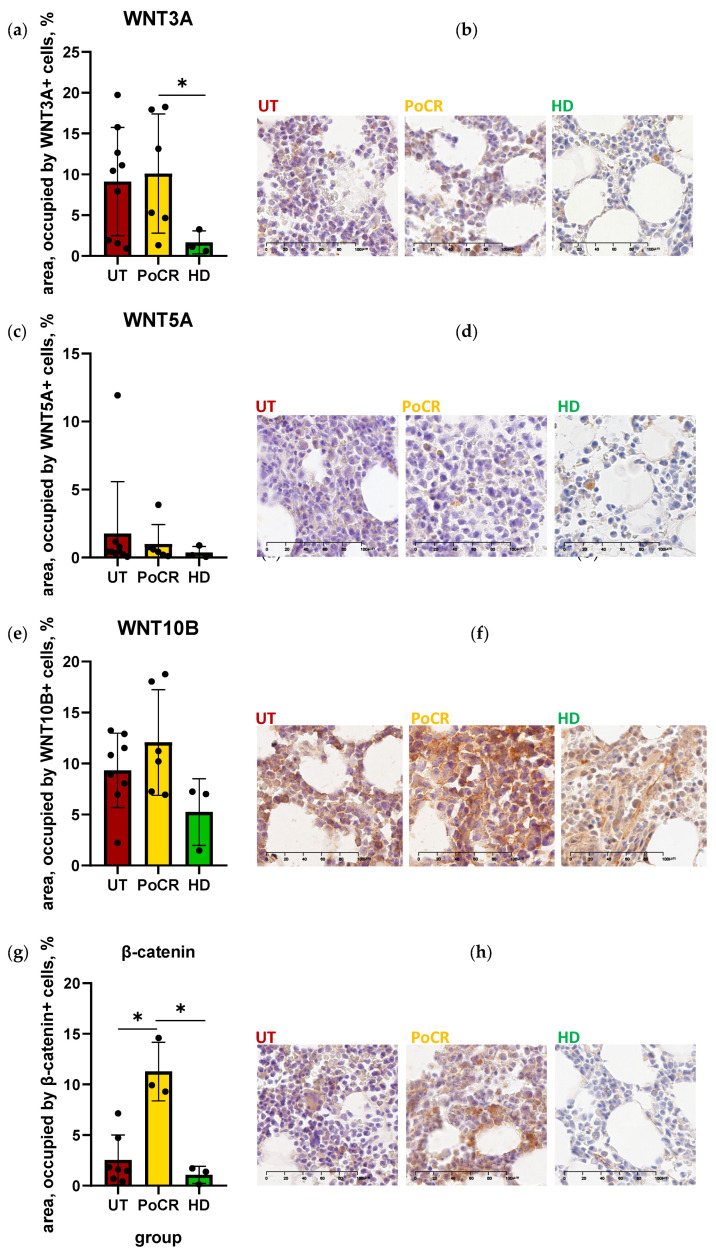
The distribution of WNT3A, WNT5A, WNT10B, and β-catenin proteins in BM trephines stained by IHC methods. Representative images (**b**,**d**,**f**,**h**) of trephines from the respective patient groups are shown for each protein. UT—primary patients; NR—patients without response to treatment; PoCR—patients with complete or partial response; HD—healthy donors. ABs (brown color) were detected by horseradish peroxidase. Nuclei were visualized with hematoxylin. The results (**a**,**c**,**e**,**g**) of the quantitative analysis of target signal distribution using Image Orbit Analysis software ver. 3.64 are presented to the left of the micrographs. The Y-axis is the area occupied by all cells stained with diaminobenzidine (peroxidase substrate) shown as the percentage of the total area. Data are represented as mean ± SD. Individual values are shown as dots. The scale bar (100 μm) is indicated in the images (**b**,**d**,**f**,**h**). * *p* < 0.05.

The percentage of area occupied by cells stained with the AB against WNT3A was increased in the majority of untreated patients (UT) and patients with partial or complete response (PoCR) compared to healthy donors (HD). The increase in WNT3A in patients with PoCR was statistically significant (Figure 2a,b).

The number of cells in which WNT5A was detected was low in all groups, ranging from 0.06 to 3.88% of the area, except a trephine of one UT patient where WNT5A-positive cells occupied 11.93% of the area (Figure 2c,d). This patient had the highest percentage of tumor cells infiltrating the BM (95%) and the del(17p)/TP53 mutation, a deletion that leads to the inactivation of the tumor suppressor p53 encoded by the TP53 gene and is a high-risk factor for MM [42].

WNT10B was identified in a relatively high number of cells across all groups, with a range from 1.47 to 18.04% of the total area. There were no statistically significant differences between groups; however, the percentage of area stained with the AB against WNT10B was higher in most specimens from MM patients than from HD (Figure 2e,f). The level of β-catenin was significantly increased in the specimens from patients with PoCR compared to both UT and HD (Figure 2g,h).

Careful examination of cell morphology showed that the above proteins were detected in both tumor cells and other cells in the trephine biopsies. Since our hypothesis was based on the importance of MSCs in establishing and maintaining the tumor niche, the next step was to test whether these proteins could be detected in MSCs in MM.

### 2.3. Distribution of WNT and β-Catenin Proteins in the BM of MM Patients

When quantifying proteins in BM trephine biopsies by IHC (Figure 2), it was difficult to determine exactly which cell type was stained positively with antibodies (ABs). We performed multiplex immunofluorescence staining of trephine biopsies using the tyramide signal amplification (TSA) method to determine in which cells of the BM the investigated proteins (WNT3A, WNT5A, WNT10B, β-catenin) were localized. An AB against CD138 was used to identify PCs and an AB against α-SMA was used to identify MSCs.

**Figure 3:**
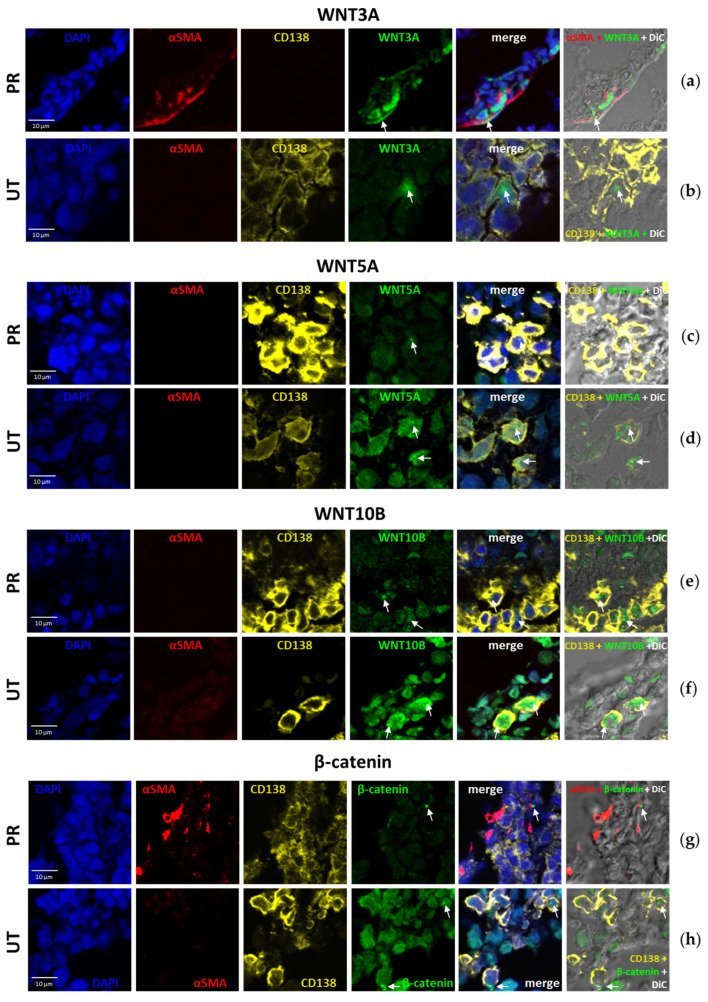
Multiplex TSA staining of BM trephine biopsies to detect WNT3A (**a**,**b**), WNT5A (**c**,**d**), WNT10B (**e**,**f**), and β-catenin (**g**,**h**) localization. Fluorescence images were obtained by confocal microscopy. DiC is an image obtained by the differential contrast method. Nuclei are stained with DAPI (blue); MSCs—with AB against α-SMA (red); PCs—with AB against CD138 (yellow); WNTs and β-catenin—with appropriate ABs (green). Merge—combined image of fluorescence signals of each band used. On the right, there are the results of combining the DiC image with the indicated fluorescent channels. The scale (10 μm) is indicated in the figure. White arrows indicate WNTs or β-catenin.

WNT3A was detected at a higher level in MSCs from patients with PoCR (38.09 ± 2.63% of total signals) (Figure 3a, Table 1), 60.44 ± 5.98% of signals were detected in unidentified cells (not stained with ABs against α-SMA or CD138), and less than 1.5% of signals were detected in PCs (Figure 3b). In contrast, in the primary enrolled patients (UT), signals were detected more frequently in PCs than in MSCs. In the trephine sample of the patient with 95% BM lesions, almost all signals from ABs against WNT3A were located in PCs, but they were relatively few in number (in 2.13 ± 0.91% of all PCs). In the trephines of patients with a lower percentage of BM lesions (30–40%), a comparable number of WNT3A signals were seen in 32.3 ± 12% of PCs, as well as in other cell types, including MSCs (7.06 ± 4.13% of all signals).

WNT5A was detectable in cells from patients in all groups, but signals were sparse. For example, PoCR patients had an average of 2.4 ± 2.31 signals per field of view (Figure 3c), while UT patients had 10.89 ± 16.04 signals (Figure 3d). The highest number of WNT5A signals was found in a patient with 95% CM lesions (68 signals in 1617 PCs (2.97% of all PCs)). Individual WNT5A signals were found in both MSCs and other niche cells.

WNT10B was detected in a higher number of cells than WNT3A and WNT5A, averaging 8.33 ± 6.15% of all cells in the field of view. An amount of 32.97 ± 10.82% of WNT10B was detected in PCs and 56.24 ± 15.07% in other cells of the niche (Figure 3e,f).

β-Catenin was also detected both in tumor cells (Figure 3h) and BM niche cells. Signals were detected in a relatively small number of cells, ranging from 1.74 ± 0.19% (UT) to 7.75 ± 1.78% (PoCR) of all cells in the field of view. Notably, β-catenin signals in PCs accounted for less than half (from 11.76% to 44.78%) of all signals; they were more frequently observed in MSCs or other cells (Figure 3g).

Thus, all of the above proteins were detected in MM tumor cells as well as in BM niche cells (including MSCs). WNT3A and β-catenin were more frequently present in MSCs or other niche cells; WNT5A and WNT10B were also found in niche cells, but their signals rarely coincided with the localization of α-SMA signals; thus, they were rarely localized in MSCs. Protein localization in some other cell types (neither CD138 nor α-SMA positive) is due to the fact that the WNT pathway is active in many BM cell types.

### 2.4. Transcriptional Activity of WNT Family Genes and the CTNNB1 Gene in BM MSCs Cultures from MM Patients

**Figure 4:**
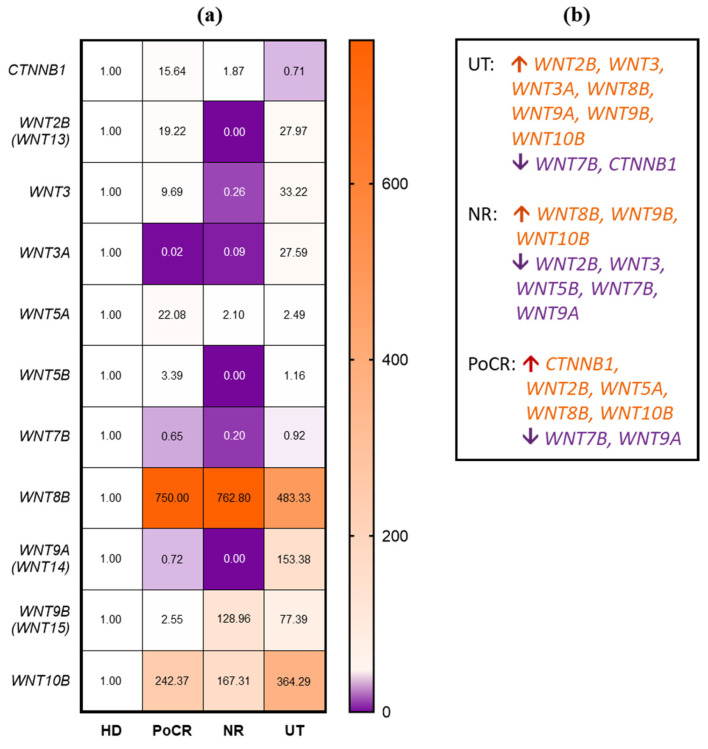
(**a**) Heatmap representing the evaluation of transcription levels of *WNT* and *CTNNB1* genes in MSC cultures by qPCR. The color scale shows the ratio to HD. In each row, the HD mRNA level for the specified gene was set as 1 (white color). The upregulated (orange) and downregulated (violet) genes for each group are shown to the right of the heatmap. (**b**) A summary of data in (**a**) expression patterns of *WNTs* and *CTNNB1* mRNAs in MSC cultures obtained from patients with different response to therapy. Orange (up arrows)—upregulation; violet (down arrows)—downregulation.

In our previous studies, we demonstrated that the MSCs of MM niche retain their tumor-associated properties during in vitro expansion [13]. We compared transcription in MSCs from HD and the following three groups of MM patients: PoCR, NR, and UT (Figure 4). *WNT10B* and *WNT8B* were strongly upregulated in all groups of patients. The expression of *WNT10B* and *WNT8B* was markedly elevated in all patient groups. The *WNT10B* gene exhibited a 364-fold increase in the UT group, a 167-fold increase in the NR group, and a 242-fold increase in the PoCR group (Figure 4). *WNT8B* exhibited from a 483- to a 762-fold increase in all patients groups (Figure 4). *WNT9A* was upregulated 153-fold in UT patients, but in other groups of patients, the downregulation was observed especially in the NR group, where transcription was very low (0–0.05-fold compared to HD). *WNT7B*, *WNT5B*, *WNT3*, and *WNT2B* were also not expressed in NR MSC grown in vitro (Figure 4). PoCR was the sole group of patients that exhibited elevated *WNT5A* (22-fold increase) and *CTNNB1* (15-fold increase) expression.

## 3. Discussion

The details of the WNT pathway regulation in MM are not yet fully investigated. It is unclear which WNT proteins are up- or downregulated to trigger these processes. Therefore, we investigated the expression of WNT family genes and the *CTNNB1* gene encoding β-catenin, a key mediator of canonical WNT signaling, in the tumor niche.

The WNT family consists of 19 genes. Differential expression was demonstrated for *WNT5A*, *WNT10B*, *CTNNB1*, *WNT1*, *WNT10A*, and *WNT5B* genes (Figure 1). The *WNT5A* gene was previously shown to be expressed in the BM of MM patients [43]. According to The Human Protein Atlas website, the *WNT10B* mRNA level is dramatically increased in MM cell lines [44], which is consistent with our data. As expected, *CTNNB1* expression is present in all groups; canonical WNT signaling via β-catenin is active in healthy individuals and is also activated in tumor cells in MM [25]. The expression levels of *WNT3* and *WNT3A* are low in all analyzed samples, although there is evidence that both products of these genes are involved in MM development. WNT3 is probably involved in the development of drug resistance of MM cells by an autocrine mechanism [45], and WNT3A may contribute to the proliferation of tumor cells [25].

We demonstrated that the WNT3A protein was expressed in MM MSCs in UT. Its gene expression was strongly upregulated in the expanded MSCs of UT patients (Figure 4). This upregulation in MSCs has not yet been demonstrated. It is one of the regulators of osteogenesis through the β-catenin-mediated canonical pathway. It has been shown that the inhibitor of the DKK1 pathway can suppress WNT3A-dependent OPG expression in osteoblasts, impairing osteogenesis [26], and artificial stimulation of WNT3A in MM can enhance osteogenesis and reduce tumor growth [41]. The involvement of WNT3A in MM development is documented. According to early data, canonical signaling is increased by WNT3A stimulation in MM cell lines but does not lead to their growth [41]; however, in other studies, WNT3A stimulates the growth of malignant PCs [25], including reducing the antitumor antiproliferative effect of lenalidomide [46]. In our study, in multiplex staining, WNT3A was detected in some UT PCs (Figure 3b), consistent with data on its role in their proliferation.

WNT5A was revealed in IHC specimens from all groups (both MM patients and HD); mostly single cells were stained, except for one UT patient with 95% BM lesion volume and poor prognosis (*TP53*/17p13 deletion causing inactivation of tumor suppressor p53). Staining percentage averaged 11.93% of the total area occupied by cells. WNT5A produced by MSCs in MM can act as a growth factor for tumor cells and increase osteoclast activity [43], which may explain the increased amount of WNT5A in this patient (although it does not explain the low levels of the protein in the other patients and in HD). However, the properties of WNT5A as an activator of osteogenic differentiation of BM MSCs are better known. It has been shown that the non-canonical pathway WNT5A/ROR2 is suppressed in MSCs as a result of contact with MM cells, but the suppression is caused not by the ligand, but by the ROR2 receptor [47]. Despite the frequent association of WNT5A with non-canonical cascades, it can also activate osteogenesis through the β-catenin-mediated canonical pathway [48]. In multiplex immunofluorescence staining, WNT5A was also detected at low levels in both PC and BM niche cells. *WNT5A* gene mRNA levels were significantly elevated in MSCs from PoCR patients. It can be hypothesized that WNT5A expression is upregulated after successful treatment, which promotes bone repair. However, the low levels of WNT5A protein in trephine biopsies remain unexplained. Further studies of WNT cascades, including tumor cell-MSC interactions, may help to clarify this issue.

WNT10B was revealed both in the TME and PCs in trephines (Figure 2 and Figure 3), and WNT10B mRNA level was high in MSC cultures in all MM groups compared to HD (Figure 4). WNT10B, like WNT5A, is responsible for the osteogenic differentiation of MSCs and osteoblastogenesis; its deficiency causes age-related bone loss and increased osteoclast activity [49]. At the same time, it is detected in osteoclasts, presumably causing the paracrine stimulation of osteoblasts [50]. WNT10B has been shown to be involved in the development of some oncohematological diseases, but its role in MM has not been studied. However, according to The Human Protein Atlas, *WNT10B* mRNA is upregulated in MM cell lines, which is consistent with the results of our bioinformatic analysis [44]. This may also explain the high mRNA levels in MSC cultures from MM patients, if they are able to produce WNT10B for the paracrine stimulation of PCs.

Canonical WNT cascades, mediated by β-catenin accumulation in the cell, normally regulate important processes in the hematopoietic niche, but in MM, they act in opposite directions. They are responsible for the balance of osteoblasts and osteoclasts in the niche, and WNT antagonists secreted by MM cells inhibit signaling and impair osteogenesis. At the same time, autocrine signaling activates the canonical WNT pathway in MM cells, promoting their proliferation and tumor progression [21]. Many FDA-approved drugs, such as antiparasitic medicines, have anti-β-catenin activity [51]; however, their use as anti-MM drugs should be evaluated based on potential benefits and risks. Though some β-catenin inhibitors, such as resveratrol, are promising candidates for combating MM progression and improving patient outcomes [52], additional research is needed to weigh the pros and cons of developing new anti-MM drugs based on β-catenin inhibitors. This is due to β-catenin’s multidirectional effects in the MM bone marrow (BM). When developing drugs in this group, the bimodal effect of β-catenin inhibitors on MM cells and their niche must be considered. We detected the most intense staining with ABs against β-catenin in trephine biopsies from patients with PoCR. This was also confirmed by qPCR data; transcription of the *CTNNB1* gene was increased in MSCs from PoCR patients compared to all other groups. Its increase compared to other groups of MM patients can be interpreted as a result of the treatment, which led to the resumption of osteogenesis processes in which β-catenin is involved. By comparing with WNT5A, we can assume that the high transcription (relative to HD transcription) in MSCs from PoCR patients is caused by the increasing intensity of osteogenic processes.

According to the results of IHC, staining with AB against β-catenin in UT patients was weak (1.74 ± 0.19%), even in trephine biopsy of the patient with 95% BM lesions. In PoCR patients the staining was more intense than in UT, although since the first studies on the role of WNT in MM it has been shown that big amount of β-catenin is present in malignant PCs, in contrast to normal PCs [25]. Bioinformatic analysis also showed no dramatic increase in its expression in PCs in myeloproliferative states. This rather unexpected result suggests that not only canonical signaling but also non-canonical cascades may play an important role in MM.

Most studies investigating the activity of the WNT pathway have been performed in cell culture. In this work, we investigated the distribution of WNT ligands and β-catenin in trephine biopsies of BM obtained from patients with MM and individuals without myeloproliferative diseases. All were found at different frequencies in trephine biopsies, both in tumor cells and in the hematopoietic niche of BM, including MSCs. We also used MSC cultures obtained from MM patient aspirates to evaluate expression at the mRNA level. The cultured cells may have different properties from cells fixed in trephine biopsies in the same position as they were in the BM in their natural environment. Nevertheless, the results of IHC and qPCR were consistent. The transcription/expression of the investigated genes and the proteins encoded by them were mainly upregulated in MM patients compared to HD (WNT3A, WNT10B, *CTNNB1*/β-catenin in the PoCR group). Although reliable differences were not found everywhere, in the bioinformatic analysis and in all experiments, we observed a change in transcription/expression. In all samples of the HD group, the values were very similar, whereas in the groups of MM patients, mRNA and protein quantity varied between samples.

The WNT cascade plays a major role in the chemoresistance of MM cells. In the present study, we identified an imbalance of the WNT cascade in both tumor cells and the tumor microenvironment (TME). An unbalanced WNT/β-catenin axis in tumor cells is one of the main causes of lenalidomide resistance [53]. Activation of the cascade by the WNT3 ligand leads to enhanced cell-adhesion-mediated chemoresistance to doxorubicin [21,54]. In situ, in our study, β-catenin was upregulated in MSCs of patients with PoCR, but not in MSCs of NR patients (Figure 4a). Trephines and MSC cultures were obtained from patients that did not receive these drugs, except for trephines from 2 PoCR patient (3 CVD courses, 1 RVD course, autoHSCT; 5 RVD), an aspirate sample from a patient with a partial response who underwent six VD courses, and one VRD, followed by autotransplantation (see Materials and Methods, Table 2 and Table 3). The treatment regimens for these patients might suggest the lenalidomide resistance.

While some WNT ligands were overexpressed in both NR and PoCR patients’ MSCs, notable differences in the mRNA levels of certain WNT genes (*WNT2B*, *WNT3*, *WNT5A*, and *WNT9B)* were observed between BM MSCs of NR and PoCR patients (Figure 4a). Human BM MSCs do not express WNT9B [55]. Therefore, its overexpression in the NR group of patients might suggest its role in chemoresistance. The data on the role of WNT2B in chemoresistance are contradictive and depend on the model. In ovarian cancer, it is associated with chemoresistance, while, in lung cancer, it is expressed at the lower level in cisplatin-resistant cells [56,57]. The role of WNT2B and WNT9B in the MM TME is underinvestigated. The WNT3 and WNT5A proteins are believed to promote chemoresistance when expressed in MM PCs. However, according to our data, they are downregulated in the BM MSC of the NR group that was resistant to treatment. The differential expression of WNT2B, WNT3, WNT5A, and WNT9B in the MSC of non-responding patients might be considered as a prognostic marker candidate in future diagnostic applications.

The results confirm that the activity of signaling cascades activated by WNT ligands is altered in the MM patients TME and show that it may not return to normal after treatment. The expression of WNT might be indicative for the recovery of the niche. For the first time, the changed status of the WNT ligand expression in the TME was demonstrated. We suggest a panel of ABs to access the TME status as well as a set of molecular markers for TME MSC culture analysis. Immunohistochemical TSA staining is a convenient diagnostic method because it is fast and can be performed in routine clinical diagnostic laboratories, while the molecular biological markers identified in MSC cultures allow for the study of the mechanisms of WNT cascade activation in cells and open new perspectives for the research and development of new therapeutic approaches.

### Limitations of the Study

The results obtained from a small statistical sample may differ from those of the general population. The number of patients in each group did not allow their response to treatment with different regimens and medicines to be analyzed. The published transcriptomes used in the study were obtained by different research groups. Although we selected studies with similar sequencing parameters, this approach may result in the use of technically different data.

## 4. Materials and Methods

### 4.1. Ethical Guidelines

Bone marrow samples were obtained from patients diagnosed with MM and from HD. Samples were collected in accordance with the Declaration of Helsinki of the World Medical Association (Declaration of Helsinki: Ethical Principles for Medical Research Involving Human Subjects, including amendments adopted at the 64th session of the WMA in Fortaleza, Brazil, October 2013) [58]. The study was approved by the Ethics Committee of the Russian Research Institute of Hematology and Transfusiology of the FMBA of Russia (Protocol No. 6-2019, dated 11 June 2019). Written informed consent was obtained from all patients and HD enrolled in the study.

### 4.2. Bioinformatics

The differential expression of the *WNT* and *CTNNB1* genes was evaluated through the analysis of raw sequencing data published by [59]. The sequencing data are deposited in European Nucleotide Archive (PRJEB37100, PRJEB36223) (https://www.ebi.ac.uk/ena, last accessed 23 October 2024). In the study referenced above, the readings were obtained through the sequencing of CD138+ PCs purified from BM samples of healthy individuals, MM patients after treatment, and MM cell lines. To remove low quality raw reads and those containing adapter sequences, the data were trimmed using Trimmomatic ver. 0.39 [60] in Paired End Mode with the parameters SLIDINGWINDOW:4:15 CROP:80 HEADCROP:10 ILLUMINACLIP:2:30:10 MINLEN:60. The quality of the resulting reads was checked with FASTQC ver. 0.12.0 [61]. The purified paired-end raw reads were mapped to the human reference genome GRCh38.p14 [62]. Mapping was performed using the HISAT2 software ver. 2.2.1 [63] with default parameters. The genome annotation file corresponding to the GRCh38.p14 assembly [64] was used to count the number of reads mapped to individual genes. The number of reads mapped to individual genes was counted using the featureCounts ver. 2.0.6 [65]. Immunoglobulin genes, mitochondrial genes, and ribosomal RNA genes were excluded from further analysis. FPKM (fragments per kilobase of transcript per million fragments mapped) [66] for individual genes was calculated, and the Z-transform of FPKM binary log values was used to construct the heat map using the package pheatmap ver. 1.0.12 [67] for R.

### 4.3. Patients

The distribution of WNT and β-catenin proteins in trephines was examined through immunohistochemical staining (IHC) using trephines from 15 patients (aged between 54 and 83 years, with a median age of 64 years). The patients were divided into three groups: first-time diagnosed UT, NR patients, and patients with PoCR (Table 2). The criteria for evaluating treatment response were based on the National Clinical Guidelines for the Diagnosis and Treatment of Multiple Myeloma [68]. Three HDs—patients without confirmed lymphoproliferative diseases—were included in the trephine biopsies study as a control group.

In the study of the transcription of *WNTs* and *CTNNB1* genes by qPCR, cells were obtained from BM aspirates. Nine patients were enrolled in the study and were divided into the same three groups as for the IHC study (UT, NR, PoCR) (Table 3). The age of the patients ranged from 59 to 70 years, with a median of 61 years. The control group (HD) consisted of three patients with a spinal cord injury who did not experience any hematopoietic disorders.

### 4.4. Collection of Trephine Biopsies and Aspirates

A sternal puncture was performed on MM patients to obtain diagnostic aspirates (1–4 mL). Aspirates from the HD were obtained via iliac crest puncture.

Prior to initiating treatment, trephine biopsies were obtained for diagnostic purposes. The material remaining after the preparation of the diagnostic specimens was utilized for the preparation of histological sections for research purposes.

The trephine biopsy specimens (paraffin blocks) and BM aspirates were provided by the Acute Leukemia Laboratory of the Research Institute of Hematology and Transfusiology of the FMBA of Russia. BM aspirates from the HD were provided by the Cell Technologies Center Pokrovsky (St. Petersburg, Russia).

### 4.5. Cell Cultures and Lines

Mesenchymal stromal cells (MSCs) were obtained from the BM mononuclear fraction. The BM aspirates were diluted with a 0.9% sodium chloride solution at a ratio of 1:3, taking into account the volume of the anticoagulant. Mononuclear cells were isolated according to the standard protocol of isolation in a Ficoll-Paque density gradient (ρ = 1.077 g/cm^3^, PanEco, Moscow, Russia). The mononuclear cell fraction was collected, diluted 1:10 with PBS, and centrifuged at 200× *g* for 10 min to remove Ficoll and platelets. The cells were then transferred to culture flasks and cultured in low glucose DMEM medium (Thermofisher, Waltham, MA, USA) supplemented with 10% fetal bovine serum (FBS) (HyClone, Logan, UT, USA), 100 U/mL penicillin, and 100 μg/mL of streptomycin (Gibco, Waltham, MA, USA). The cells were incubated at 37 °C in an atmosphere of 5% CO_2_ and 7% O_2_ (“physiological hypoxia” is the cultivation of cells at an oxygen concentration equivalent to the tissue oxygen concentration) [69]. The medium was changed every 3 days. Upon reaching a density of 70–80%, the cells were harvested using trypsin and Versen solutions (Gibco, Waltham, MA, USA) and were split at a ratio of 1:2–1:3. MSCs at passages 1–5 were used for further experiments.

### 4.6. Oligonucleotides

The primers (Evrogen, Moscow, Russia) used for the amplification of 10 *WNT* family genes, as well as the *CTNNB1* gene [70], are listed in Table 4.

To amplify the reference *GAPDH* gene, the primer pair 5′-AGGTCGGAGTCAACGGATTT-3′ (forward) and 5′-TTCCCGTTCTCTCTCAGCCTTGAC-3′ (reverse) [71] was used.

### 4.7. RNA Isolation and cDNA Synthesis

Total RNA was isolated from MSCs of HD, UT, NR, and PoCR using a monophasic aqueous solution of phenol and guanidine isothiocyanate (ExtractRNA reagent, Evrogen, Moscow, Russia). After the cells were lysed with ExtractRNA, 0.2 mL of chloroform per 1 mL of reagent was added and centrifuged to separate the phases. The upper aqueous phase containing RNA was then aspirated, and 100% isopropanol was added. Subsequently, the supernatant was removed, and the RNA pellet was washed with 75% ethanol, dried, and then dissolved. The RNA was further purified from DNA contamination through incubation with DNase I. The reaction was terminated through the addition of EDTA and heating.

The purified RNA was used as a matrix in reverse transcription reaction using M-MuLV-RH enzyme (Biolabmix, Novosibirsk, Russia) and oligo(dT)16 primers.

### 4.8. Real-Time PCR

Real-time PCR was conducted using a CFX96 real-time system (Bio-Rad, Hercules, CA, USA). A 5× qPCRmix-HS SYBR reaction mix (Eurogen, Moscow, Russia) containing Taq polymerase, SYBR Green I dye, deoxynucleoside triphosphate mixture, Mg^2+^, and PCR buffer was used. Single-stranded cDNA was used as the matrix.

The thermocycling conditions were as follows: an initial denaturation at 95 °C for 5 min; then 40 cycles at 95 °C for 10 s (denaturation), 56 °C for 30 s (annealing), and 72 °C for 20 s (3-step protocol). At the end of the program, a melting curve step was added consisting of heating from 65 °C to 95 °C in 0.5 °C increments.

### 4.9. Immunohistochemistry (IHC)

Trephine biopsy samples were fixed in 10% buffered formalin (Biovitrum, Saint Petersburg, Russia) and were decalcified for three days in 14% EDTA solution at 37 °C. Dehydration and paraffin embedding were performed according to the standard method in an automatic histological processor (Diapath, Martinegro, BG, Italy) using dehydrating solution IsoPREP (Biovitrum, Saint Petersburg, Russia) and a paraffin medium HISTOMIX (Biovitrum, Saint Petersburg, Russia). Sections (2–3 µm) were cut using an HM325 rotary microtome (Thermofisher, Waltham, MA, USA).

The distribution of WNT3A, WNT5A, WNT10B, and β-catenin proteins was assessed by staining BM trephine sections with the corresponding ABs (ABclonal, Woburn, MA, USA).

For IHC, sections (2 μm) were cut and mounted on poly-L-lysine slides. Sections were cleared in xylene and rehydrated by passing them through a series of alcohols. Antigen retrieval was performed in a PT-link water bath (Dako, Glostrup, Denmark) at 98 °C in pH 9.0 buffer (Dako, Glostrup, Denmark) for 25 min. To block endogenous peroxidase, EnVision FLEX Peroxidase Blocking (Dako, Glostrup, Denmark) was applied to sections for 10 min at room temperature in a humidified chamber. The sections were incubated with a panel of primary ABs (Table 3) for 16–18 h at +4 °C in a humidified chamber. The ABs were diluted in Emerald diluent (Cell Marque, Rocklin, CA, USA). Visualization was performed using the N-Histofine^®^ Simple Stain MAX PO (Multi) detection system (Nichirei Biosciences, Tokyo, Japan); incubation was carried out in a humidified chamber at 37 °C for 30 min. Sections were washed from the ABs in EnVision FLEX wash buffer (Dako, Glostrup, Denmark). Diaminobenzidine (DAB) chromogen (Roche, Basel, Switzerland) diluted in 1 mL of Substrate Buffer (Dako, Glostrup, Denmark) was used to visualize the reaction product, and the incubation time was 8–10 min. For nuclear staining, the sections were immersed in Mayer’s hematoxylin (ErgoProduction, Saint Petersburg, Russia) for 30–45 s. The sections were then dehydrated in three changes of isopropyl alcohol and cleared in two changes of xylene, after which they were mounted under coverslips in synthetic mounting medium Vitrogel (ErgoProduction, Saint Petersburg, Russia).

The negative control was performed by replacing the primary ABs with TBS buffer.

### 4.10. Multiplex Fluorescence IHC

The localization of WNT3A, WNT5A, WNT10B, and β-catenin ligands in specific cell types of BM trephine biopsies was investigated using Opal™ 4-Color Automation IHC Kit 50 slides (Akoya Biosciences, Marlborough, MA, USA). ABs against CD138 and Alpha Smooth Muscle Actin (α-SMA) (both from Diagnostic BioSystems, Pleasanton, CA, USA) were used to identify PCs and MSCs, respectively.

The method is based on the principle of TSA, with the advantage of reducing the probability of non-specific binding [72]. Primary ABs, secondary ABs conjugated to HRP, and Opal fluorochrome were added sequentially. A fluorochrome was not bound to the ABs that were removed after each cycle. In contrast, a fluorochrome bound covalently to tyrosine residues of cell proteins, remaining on the sample in close proximity to the AB-binding site. The staining cycle was then repeated with another primary AB.

The deparaffinization, rehydration, and antigen retrieval steps were conducted as described above for IHC. Following the peroxidase block, the sections were incubated with the supplied buffer for 10 min to block non-specific binding. A primary AB (against one of the ligands tested), a secondary AB (Opal Polymer HRP Ms + Rb), and Opal fluorochrome were then applied according to the manufacturer’s protocol. The primary and secondary ABs were removed by heating in a water bath at 98 °C for 25 min. The steps were repeated, starting from the peroxidase block step. The following staining cycles were performed for each section:

Step 1: α-SMA AB (Opal 690 fluorophore) to label MSCs;

Step 2: CD138 AB (Opal 520 fluorophore);

Step 3: An AB against one of the following antigens: WNT3A, WNT5A, WNT10B, β-catenin (Opal 570 Fluorophore).

After the application of all ABs, the sections were incubated with DAPI to visualize nuclei. Slides were then briefly rinsed in ethanol, air-dried, and embedded in medium under coverslips.

### 4.11. Microscopy

Trephines were examined after multicolor immunofluorescence staining using an Olympus FV3000 confocal laser microscope (Olympus Corporation, Tokyo, Japan). The lasers used were 488 nm for Opal 520, 561 nm for Opal 570, 640 nm for Opal 690, and 405 nm for DAPI. Scanning was performed at an optical section thickness of 0.8 μm. Photos were taken at 400x microscope magnification. The MATL function was used to scan and stitch multiple images. For each slide, images of at least three random visual fields were taken.

Post IHC slides were scanned using a KFBio Magscanner KF-PRO-005-Ex. Further analysis was performed on digitized images of the sample. Areas were highlighted at 40× approximation, corresponding to 40× microscope magnification, and 10 areas were counted for each sample. Morphometric evaluation of the results was performed using K-Viewer and Orbit Image Analysis software ver. 3.64 [73]. When analyzing the IHC results, the area (in %) occupied by brown-stained (DAB) cells was calculated in relation to the area occupied by all cells in the trephine (excluding adipocytes and megakaryocytes) using Orbit Image Analysis ver. 3.64.

### 4.12. Statistical Analysis

The significance of differences (*p* < 0.05) for the results of the bioinformatic analysis, IHC, and qPCR was assessed by a Kruskal–Wallis one-factor analysis of variance (when comparing three or more samples) and a Mann–Whitney U-criterion (when making pairwise comparisons) using GraphPad Prism ver. 8.0.0 for Windows (GraphPad Software, San Diego, CA, USA). Data are presented as mean ± SD. Prior to statistical processing, qPCR results were normalized using the ∆∆Ct method.

## 5. Conclusions

The expression of WNT family ligands as well as the canonical intracellular mediator of the WNT pathway, β-catenin, differs in MM patients compared to HD and also differs between treated and untreated patient groups. For the first time, we show differences in the expression of WNT3A, WNT5A, WNT10B, and β-catenin ligands in patient trephine biopsies. Multiplex IHC staining showed that the listed proteins were localized in tumor cells as well as in cells of the BM hematopoietic niche, including MSCs. Expression also differed at the level of mRNA isolated from patient BM MSC cultures (for *WNT3A*, *WNT5A*, *WNT10B*, *WNT8B*, *CTNNB1*, and other genes). The data obtained can be used for diagnostic purposes to assess the state of the hematopoietic niche of MM patients and to predict the response to therapy.

## Figures and Tables

**Table 1:** Distribution of WNT and β-catenin proteins in the BM of MM patients.

	UT *						PoCR					
	MSCs		PCs		Others		MSCs		PCs		Others	
	Stained Cells, %	Signals per 3 Fields of View	Stained Cells, %	Signals per 3 Fields of View	Stained Cells, %	Signals per 3 Fields of View	Stained Cells, %	Signals per 3 Fields of View	Stained Cells, %	Signals per 3 Fields of View	Stained Cells, %	Signals per 3 Fields of View
**WNT3A**	11.75 ± 7.27	10	17.09 ± 18.92	94	0	41	36.23 ± 5.7	223	60.97 ± 10.7	15	0	356
**WNT5A**	5.09 ± 4.49	19	1.24 ± 2.56	77	0	39	3.94 ± 2.77	5	2.72 ± 0.59	4	0	2
**WNT10B**	21.5 ± 5.58	6	15.74 ± 4.92	12	0	28	-	1	21.64 ± 2.67	46	0	122
**β-catenin**	26.68 ± 8.21	35	1.24 ± 2.02	22	0	49	54.55	6	5.10 ± 2.62	30	0	159

* PR—patient with partial response; UT—untreated patient.

**Table 2:** Medical history of trephine biopsy donors.

Group	Age	Type of Response *	M-Protein Type	BM Infiltration, % (Pre-Treatment)	Myelogram, % PCs (Pre- and Post-Treatment)	Karyotype	Therapy **
PoCR PCs < 10% (*n* = 6)	65	VGPR	IgG kappa	40	Before: 43.6 After: 0	?	4 CVD courses, 6 DaraRd courses
	61	VGPR	IgG lambda	20–30	After: 2.2	13q14 and 13q34 deletions	3 CVD courses, autoHSCT
	59	PR	IgG kappa	60–70	Before: 8.8 After: 5.6	?	3 CVD courses, 1 RVD course, autoHSCT
	69	PR	IgA kappa	70–80	?	?	5 VRD courses
	64	PR	IgA kappa	Single cells	After: 2.8	No abnormalities	autoHSCT
	54	SD	- (non-secretory MM)	10	After: 6	?	4 CVD courses
NR (*n* = 1)	61	SD	IgA kappa	50	After: 14.4	No abnormalities	3 VCD courses, autoHSCT
UT (*n* = 8) (Trephine biopsies: *n* = 9 (2 trephine biopsies were taken from one patient))	83	-	IgG lambda	15	?	?	-
	83	-	IgG lambda	30–40	46.8	?	-
	72	-	IgA kappa	30–40	21.6	?	-
	71	-	IgG kappa	95	42.6	17p/TP53 deletion	-
	58	-	IgA kappa	70–80	?	?	-
	60	-	?	90	?	?	-
	70	-	IgG lambda	80–90	36.4	?	-
	56	-	- (non-secretory MM)	80	6	?	-

* Types of response: PoCR—partial or complete response; VGPR—very good partial response; PO—partial response; SD—disease stabilization; NR—no response; UT—untreated. Unknown data are marked with “?”. ** Regimen abbreviations: CV = cyclophosphamide–bortezomib; CVD = cyclophosphamide–bortezomib–dexamethasone; VCD = bortezomib–cyclophosphamide–dexamethasone; VRD = bortezomib–lenalidomide–dexamethasone; RVD = lenalidomide–bortezomib–dexamethasone; DaraRd = daratumumab–lenalidomide–dexamethasone; autoHSCT—Autologous Hematopoietic Stem Cell Transplantation.

**Table 3:** Medical history of aspirate donors.

Group	Age	Type of Response *	M-Protein Type	BM Infiltration, % (Post-Treatment)	Myelogram, % PCs (Post-Treatment)	Karyotype	Therapy **
PoCR PC < 10% (*n* = 3)	61	CR	IgG lambda	1–2	1	No abnormalities	5 VCD courses, autoHSCT
	59	CR	IgG kappa	1–2	2.6	No abnormalities	6 VD courses
	59	VGPR	IgG lambda	1–2	2.8	No abnormalities	VRD, autoHSCT
NR (*n* = 3)	50	SD	IgG kappa	50	47	14q32 monosomy, 13q14 and 13q34 deletions, TP53/17p13 deletion or monosomy	6 VD courses, autoHSCT
	61	SD	IgA kappa	50	14.4	No abnormalities	3 VCD courses, autoHSCT
	70	SD	IgG lambda	90	82.4	Y chromosome loss	2 VCD courses
UT (*n* = 3)	64	-	?	?	66	?	-
	?	-	IgG kappa	?	?	?	-
	?	-	?	?	?	?	-

* Types of response: abbreviations are the same as in Table 2. ** Regimen abbreviations: VD = bortezomib–dexamethasone. The other abbreviations are the same as those in Table 2. “?”—no data in the medical history.

**Table 4:** Primer sequences used in this study.

Gene	Forward Primer	Reverse Primer
*WNT3*	5′-GGAGAAGCGGAAGGAAAAATG-3′	5′-GCACGTCGTAGATGCGAATACA-3′
*WNT3A*	5′-CCTGCACTCCATCCAGCTACA-3′	5′-GACCTCTCTTCCTACCTTTCCCTTA-3′
*WNT5A*	5′-GAAATGCGTGTTGGGTTGAA-3′	5′-ATGCCCTCTCCACAAAGTGAA-3′
*WNT5B*	5′-CTGCCTTTCCAGCGAGAATT-3′	5′-AGGTCAAATGGCCCCCTTT-3′
*WNT7B*	5′-CCCGGCAAGTTCTCTTTCTTC-3′	5′-GGCGTAGCTTTTCTGTGTCCAT-3′
*WNT8B*	5′-TCCCAGAAAAACTGAGGAAACTG-3′	5′-AACCTCTGCCTCTAGGAACCAA-3′
*WNT10B*	5′-CTTTTCAGCCCTTTGCTCTGAT-3′	5′-CCCCTAAAGCTGTTTCCAGGTA-3′
*WNT2B* (previously *WNT13*)	5′-TGCCAAGGAGAAGAGGCTTAAG-3′	5′-GTGCGACCACAGCGGTTATT-3′
*WNT9A* (previously *WNT14*)	5′-CTTAAGTACAGCAGCAAGTTCGTCAA-3′	5′-CCACGAGGTTGTTGTGGAAGT-3′
*WNT9B* (previously *WNT15*)	5′-CAGGTGCTGAAACTGCGCTAT-3′	5′-GCCCAAGGCCTCATTGGT-3′
*CTNNB1*	5′-CTGCTGTTTTGTTCCGAATGTC-3′	5′-CCATTGGCTCTGTTCTGAAGAGA-3′

## Data Availability

Data are contained within the article. The raw sequencing data for the bioinformatic analysis of the *WNTs* and *CTNNB1* genes’ differential expression were taken from the article, published by Emde-Rajaratnam et al. in 2023 [59]. These data are deposited in the European Nucleotide Archive (PRJEB37100, PRJEB36223) (https://www.ebi.ac.uk/ena, last accessed 23 October 2024).

## Abbreviations

BM	bone marrow
MM	multiple myeloma
UT	primary patients
NR	patients without response to treatment
PoCR	patients with complete or partial response
HD	healthy donors
MSCs	mesenchymal stromal cells
PC	plasma cells
TME	tumor microenvironment
